# Cases *of Injuries of the Head, with Observations*

**Published:** 1800-01

**Authors:** John Chapman

**Affiliations:** Surgeon, Ampthill


### CASES *of Injuries of the Head, with Obſervations, by* JOHN CHAPMAN, *Surgeon, Ampthill.*

THE fondneſs for trepanning, ſo much inculcated by Mr. Pott, and ſo very anxiouſly ſupported by Mr. Benjamin Bell, has juſtly met with two very able antagoniſts in Mr. John Bell* and Mr. Abernethy,† the latter having given ſix caſes of fracture, with depreſſion, that recovered without the uſe of the trephine: in theſe caſes no ſubſequent inconvenience was occaſioned by the depreſſion. Mr. Latta has alſo given two caſes of depreſſion that recovered where the bone, as he ſuppoſes, recovered its form by its own elaſticity; in theſe two caſes there could not exiſt any fracture. For my own part, I have never ſeen any caſe of this kind of bending in the bones of the cranium, yet can readily believe that it may frequently be the caſe in young children. I have met with two caſes (in ſuch ſubjects) where the bones of the fore arm were bent very conſiderably, without any fracture, and that required conſiderable force to ſtraighten them.

The following Caſes ſtill further confirm Mr. Abernethy's opinion, that there are many inſtances of fractures with depreſſion, that will do perfectly well without the uſe of the trephine, the brain becoming accuſtomed to the preſſure.

CASE I.—A child four or five years old, the daughter of one

---

* Diſcourſes on Wounds, part ii. p. 153. † Surgical Eſſays, part iii.

of the nurfes, was admitted into St. George's Hofpital under Mr. Walker, having received a blow upon the left parietal bone, which was followed by extravafation; upon examination it gave the feel of fracture with depreffion, (of the exiftence of which there was not any doubt,) but as there were no fymptoms or inconvenience attending this preternatural preffure, Mr. W. did not think it prudent to divide the fcalp, but directed cloths, wet with a folution of muriated ammonia in vinegar and water, to be applied to the part, which was continued for a fortnight, till the extravafation had entirely fubfided, the depreffion ftill remaining. The child was kept in the hofpital ten days or a fortnight longer, and as fhe remained perfectly well, was difcharged. I heard a confiderable time after this, that fhe felt not the leaft inconvenience.

CASE 2.—A. S. of Silfoe, a woman about twenty years of age, fays, that feven years ago, being at play with her brothers, fhe had the misfortune to fall backwards upon the floor; fhe immediately perceived a fwelling a little above the occipital ridge, which fubfided in a fhort time without any other fymptoms; fince this, fhe has been at different times affected with an acute pain in her head, which, fhe fays, always originates in that part. About three years ago fhe had feveral very ftrong epileptic fits, which have very rarely returned fince, but fhe is now much troubled with pain in her head and vertigo. On examining the part affected, I difcovered a triangular depreffion of the cranium, which fhe never recollects to have given her the leaft pain upon preffure being applied to it.

Mr. Cruikfhank, in his Lectures, relates the cafe of a woman that recovered from an apoplexy in two or three days, without any remaining paralytic affection, and was apparently well, remained fo for two years, and then died of a fever. On opening the head betwixt the *dura* and *pia mater*, was found a cyft, the parietes of which were formed of coagulable lymph, containing a quantity of fluid blood. This cyft is preferved in the Mufeum in Windmill-ftreet.

Mr. John Bell, fays, "I have very often feen the remains of moft unequivocal depreffions of the fkull, (e. g.) from the kick of a horfe in boys who have grown up, (the depreffion ftill continuing) till they became ftrong and healthy men."*

There are alfo cafes where the injury is entirely confined to the fcalp, which, by the fymptoms and feel of the part, give every appearance of depreffion; but by waiting a few days, the alarming fymptoms, extravafation, and feel of depreffion have difappeared.

* J. Bell's Difcourfes on Wounds, part ii, p. 138.

diſappeared. Theſe caſes require a ſuperior ſagacity, or tactus eruditus, to diſcover whether fracture does or does not exiſt, previous to dividing the ſcalp, and I can readily conceive, that even our firſt ſurgeons might ſometimes be diſappointed.

Case 3.—T. O. of Ampthill, a boy about thirteen years old, on running down the ſtreet, his feet ſlipped, and he ſtruck his head againſt a ſtone; a conſiderable ſwelling, with extravaſation, immediately appeared upon the anterior part of the parietal bone, giving the exact feel of fracture with depreſſion. The boy became very ſtupid and drowſy, was frequently ſick. The ſickneſs and drowſineſs increaſed in the evening, his pulſe became more full and quick, with thirſt, &c. I wiſhed now to bleed him, but it was not permitted, and nothing more was done than a lotion of muriated ammonia applied to the part. On the following morning he was much the ſame, a purge was exhibited, and as ſpeedily rejected; the ſaline mixture with ſal polychreſt was then had recourſe to, and continued during the day and night. On the third day, had ſlept quietly, the ſickneſs gone off, had two ſtools, and was conſiderably better; the extravaſation and feel of depreſſion remained the ſame, continued ſome days, then gradually ſubſided, leaving a longitudinal ridge under the ſcalp, which is not now, after an elapſe of three weeks, entirely removed. The following caſe alſo ſhews how much we may be deceived by theſe appearances.

Case 4.—A ſervant man belonging to Mr. P——, of Maulden, received a kick from a horſe over the orbital ridge, which divided the integuments down to the bone; the force of the blow knocked the man backwards, and he fell with the back of his head on a large pebble; he was taken up ſenſeleſs, the wound was properly dreſſed and attended to; the bone had not received the leaſt injury; he was let blood copiouſly, proper medicines were given, and he was put to bed: the next day the ſtupor and drowſineſs much the ſame, complained occaſionally of his head, his pulſe 90 and full; aperients were given, and venæſection was repeated. On the third day he had a very reſtleſs night; his pulſe quicker, more thirſt, ſometimes ſick, and except occaſional intervals of coma, was very reſtleſs; the aperients had operated very well. A bliſter was applied betwixt the ſhoulders, and diaphoretics were given. On examining the head this day, a little ſwelling was diſcovered on the poſterior part of the parietal bone, occaſioned by the fall, which gave the feel of fracture with depreſſion; nor was I ever more diſappointed in my life, than in freely laying open the ſcalp, to find that no fracture exiſted, and that this feel had been occaſioned by extravaſated blood; in dividing the ſcalp a ſmall artery was divided, and allowed to bleed for a little while; the wound was dreſſed, dreſſed, preſſes were given, and the diaphoretics repeated; but he became more reſtleſs, and on the fourth day he died. I regret that I could not procure leave to open his head. The firſt day or two the ſymptoms were evidently thoſe of concuſſion, that rapidly changed to inflammation. According to Mr. Benjamin Bell, I ſhould have been warranted in the uſe of the trephine, (as there was a ſuppoſition of extravaſation) in ſearch of blood or matter oppreſſing the brain;* but this, in my opinion, would have been extremely raſh; the idea of it brings the following caſe to my recollection.

Case 5.—A labouring man, about ſixty years of age, in lopping a tree his foot ſlipped, and in his fall ſtruck the bill or hatchet he held in his hand againſt his head, and divided the ſcalp upon the frontal bone. A ſurgeon was ſent for; on examining the wound, he declared the ſkull to be fractured; the next morning (being in partnerſhip) himſelf and partner arrived, with the intention of applying the trephine; the wound was dilated to a conſiderable extent, and an inconſiderable fiſſure † was diſcovered. One of the ſurgeons inſiſted upon it, that it was a compleat fracture; the reply of the other was, if you will ſay it is, I will trepan him. The man during all this time was in his perfect ſenſes, without a ſingle ſymptom of a compreſſed brain. A gentleman preſent begged they would poſtpone the operation; they now attempted to ſcrape out the fiſſure with the raſpatory, which was effectually done with little trouble. The man recovered very well.

Every man, previous to applying the trepan, ought to aſk himſelf, for what he is going to trepan? "To think that a fractured ſkull is a chief cauſe, or even an abſolute ſign of danger, is a very erroneous notion; it is not the damage done to the ſkull, but the injury to the brain, that is the cauſe of danger; and the fracture of the ſkull is but a faint, uncertain mark of the harm done to the brain." ‡ Again, "There is ſtill but one motive for applying the trephine, viz. to relieve the brain from compreſſion." §

Now I am ſpeaking of affections of the brain, I cannot forbear obſerving, that I have long been diſſatisfied with the Edinburgh

* Syſtem of Surgery, Vol. III. p. 121.

† "A fiſſure is not of itſelf a motive for trepanning the ſkull; but if with a fiſſure the patient lies oppreſſed, then the oppreſſion is the mark of danger, perhaps, from extravaſated blood; and the fracture or fiſſure of the ſkull, marks the point on which we ſhould apply our trepan." J. Bell's Diſcourſes on Wounds, Part II. p. 145.

‡ J. Bell's Diſcourſes on Wounds of the Head, p. 137.

§ Ibid, p. 144.

burgh treatment of concussions of the brain, viz. with cordials, wine, and stimulants; this appears to be uniformly the same with Mr. B. Bell,* Mr. J. Bell,† and Mr. Latta,‡ and is directly opposite to the experience of Pott. My ideas on this subject are so exactly consonant to what has been said by Mr. Abernethy, § that I shall therefore refer my readers to his Essays. In concussion of the brain, (in Hunterian terms) we ought to take great care to keep up a proper equilibrium between the powers of the part and their action, or we shall have more inflammation than may be consistent with life, it therefore requires the greatest caution in the use of stimulants; for whilst concussion may be subsiding on the one hand, inflammation may be making rapid strides on the other, which was clearly the case in Case the 4th. I shall beg leave to subjoin the following instances of concussion without any comment.

CASE 6.—Mr. T. grocer, of Leighton Buzzard, in reaching down some candles, his foot slipped, and the back of his head struck against some drawers; he was taken up senseless; in a short time was somewhat recovered, complained much of his head, was very giddy, and talked incoherently. I did not see him for two or three hours after the accident, his pulse was beating full and strong; I immediately took away twelve ounces of blood, and directed salines with antimonial wine and tinct. opii; the next morning he still complained of his head, was very giddy, talked very wandering, his pulse too quick and full; I gave a saline aperient, which operated very well; repeated the salines in the evening; the day following he was more sensible, but continued a little wandering, with occasional loss of memory for two or three days, and then recovered without any farther inconvenience.

---

### *Mr. Christie's Observations on Gun-shot Wounds.*

[ Continued from our last Number, pp. 447. ]

A Private, of the name of Doogen, belonging to the 27th regiment, was shot by a musquet ball entering half way between the right breast and axilla, where the ribs sit pretty close on each

* System of Surgery, Vol. III. p. 141.

† On Wounds of the Head, p. 139.

‡ System of Surgery, Vol. II. p. 177, 178.

§ Surgical Essays, Part III. p. 59, 60, &c.

